# Validation of a New Instrument for Self-Assessment of Nurses' Core Competencies in Palliative Care 

**DOI:** 10.1155/2014/615498

**Published:** 2014-07-16

**Authors:** Kari Slåtten, Ove Hatlevik, Lisbeth Fagerström

**Affiliations:** ^1^Lovisenberg Diaconal University College, Oslo, Norway; ^2^National Centre for ICT in Education, Oslo, Norway; ^3^Faculty of Health Sciences, Buskerud and Vestfold University College, Papirbredden, Grønland 58, 3045 Drammen, Norway; ^4^Åbo Akademi University, Vaasa, Finland

## Abstract

Competence can be seen as a prerequisite for high quality nursing in clinical settings. Few research studies have focused on nurses' core competencies in clinical palliative care and few measurement tools have been developed to explore these core competencies. The purpose of this study was to test and validate the nurses' core competence in palliative care (NCPC) instrument. A total of 122 clinical nurse specialists who had completed a postbachelor program in palliative care at two university colleges in Norway answered the questionnaire. The initial analysis, with structural equation modelling, was run in Mplus 7. A modified confirmatory factor analysis revealed the following five domains: knowledge in symptom management, systematic use of the Edmonton symptom assessment system, teamwork skills, interpersonal skills, and life closure skills. The actual instrument needs to be tested in a practice setting with a larger sample to confirm its usefulness. The instrument has the potential to be used to refine clinical competence in palliative care and be used for the training and evaluation of palliative care nurses.

## 1. Introduction

With populations aging, more persons are living with the effects of serious illness. Persons with incurable illness need clinical assessments and care from a wide range of healthcare services [1, 2]. The demand for skilled services to help individuals with incurable and life-limiting diseases and their families will increase in the coming years [3]. Palliative care in Norway has been closely integrated with cancer care units; therefore, palliative care units and their specialists remain limited in number [4]. A large proportion of patients in Norway die in their community, either at home or in nursing homes [4, 5]. To improve the quality of palliative end-of-life care, clinicians' competence has been emphasized for some years, particularly in regard to the assessment of symptoms [5]. A clear need exists for a validated instrument for use in assessing nurses' competence in palliative care.

To ensure optimal care and treatment, at least a basic level of competence in palliative care nursing is needed [6–8], but the complexity of palliative care at end-of-life requires advanced clinical care competence [2, 9–12]. Nurses' clinical competence in symptom management and application of best care practices is crucial [13]. Competence is a prerequisite for high quality nursing in clinical settings [14].

A more systematic assessment of nurses' palliative care competence is considered to be an important research area [9, 12]. To the best of our knowledge, the various domains of competence particular to palliative care nursing and the assessment of nurses' competence in this area have not been previously explored in Sweden, Finland, Denmark, Iceland, or Norway.

In 2007 a new instrument measuring nursing clinical competence in palliative care was developed and pilot-tested among nurses who had graduated from a post-bachelor, palliative care specialist program. The study associated with this project had a cross-sectional design and was the first Nordic survey of nursing clinical competence in palliative care [13]. The study showed that competencies in managing pain, nausea, anxiety/restlessness, fatigue and dryness of mouth as well as time available for nursing care, measured using the Edmonton symptom assessment system (ESAS), had a positive effect on care routines.

According to Newble [14], competence can be seen as a prerequisite for performance in the real clinical setting. Competence in palliative care can be considered from a common viewpoint, with a focus on knowledge, key skills, personal qualities, attributes and behaviours, which Becker [8, 15–17] terms “core competences.” According to Becker [8], an individual must have core competencies in order to perform a job effectively. The European Association for Palliative Care [18] (EAPC) has defined the following core competencies for health- and social-care professionals: autonomy, dignity, relationship between patient and healthcare professionals, quality of life, position towards life and death, communication, public education, multiprofessional approach, and grief and bereavement. Murray [19] described three general care patterns in palliative care approaches: (a) supported self-care, (b) episodic disease management, and (c) case management. Seymour [20] stated that the key terms in palliative care nursing are teamwork, the relief of suffering, the promotion of quality of life and hope, knowing the patient and the promotion of dignity, comfort, and support. According to Becker's analyses of earlier research, the core categories of palliative care nurses' clinical competencies are communication skills, psychosocial skills, teamwork skills, physical care skills, life closure skills, and intrapersonal skills. According to Connolly et al. [21], many palliative care competence frameworks fail to indicate how the framework could inform curriculum development or support continued professional development and life-long learning in clinical practice. We understand from these references that good clinical skills in alleviating a patient's suffering and “knowing the patient” and her/his preferences and needs are central core competencies in palliative nursing and that productive teamwork is needed for promoting the patient's quality of life in the palliative phase.

Measuring nurses' competence in clinical practice can be done in different ways [22–24]. Questionnaires for the self-assessment of nurses' clinical competence in palliative care are lacking. Ross et al. [25] developed the palliative care quiz for nurses (PCQN), and Adriaansen et al. [26, 27] later supplemented and modified the PCQN for Dutch use before and after palliative care courses. However, these instruments were not suitable for our purpose because they did not include content related to symptoms or life closure skills, both recommended as necessary in earlier research and delineated in national recommendations in Norway. Therefore the purpose of this study was to test and validate a new instrument, the Nurses' Core Competence in Palliative Care (NCPC) instrument.

## 2. Methods

### 2.1. Participants and Data Collection

The total study population was comprised of 235 clinical nurse specialists and former students who had completed a postbachelor program in palliative care at two university colleges in Norway between 2000 and 2006. The questionnaire was sent to the study participants' home addresses. A letter with a reminder about the survey was sent to those who did not respond the first time, which led to 10 additional responses. The final sample included 122 participants (51.9% response rate).

### 2.2. The NCPC

First developed in 2007, the original NCPC questionnaire was used to evaluate nurses' core competence in palliative care. The original questionnaire and material, which was validated in this actual study, consisted of 176 questions including questions to identify nursing competency in palliative care, background variables, students' experiences of their postbachelor's program, and dichotomous questions about their experiences and attitudes. As a first step we shortened the questionnaire by excluding background variables and other extraneous questions, for example, about experiences of the postbachelor program and various dichotomous questions not suitable for our factor analysis. As a second step, a theoretical evaluation occurred. By analysing existing literature on clinical competency in palliative care from earlier international research and literature in general [6, 7, 13, 27–29], in particular Becker's analysis of the core competencies in palliative care [15–17], we determined that five competence domains were appropriate in the instrument. The third step had a more explorative approach and was based on the domains derived during the second step. The aim of the third step was to analyse the questions in order to identify the items and domains most suitable for an instrument that measures clinical competency in palliative care. A combination of repeated factor analysis and analysis of consistency, using Cronbach's alpha, was applied for those questions revealed during the theoretical analyses as seeming potentially interesting. Overall, these analyses revealed the items that matched and created a concept. In the fourth step, the process of exploring the items and domains, we took into account the ratio between the numbers of items and the number of participants in the study. To be able to conduct factor analysis, the number of items and domains had to be proportionate to the number of study participants [30]. We therefore considered it important not to split the material into separate parts during the analysis.

The final NCPC questionnaire consisted of 26 questions covering the following five core competence domains.

*Knowledge in Symptom Management*. Five items were used to measure this domain: dealing with pain, nausea, anxiety/restlessness, fatigue and dryness of mouth.

*Systematic Use of the Edmonton Symptom Assessment System (ESAS)*. Six items were used to measure this domain: the use of ESAS in dealing with pain, nausea, anxiety/restlessness, fatigue, dryness of mouth, and wellbeing when breaking bad news.

*Teamwork Skills*. Five items were used to measure this domain: collaboration in dealing with pain, nausea, anxiety/restlessness, fatigue, and dryness of mouth. Intercollegial collaboration could occur between professionals in the same or separate disciplines or professionals in the same or separate services.

*Interpersonal Skills*. Three variables were used to measure this domain: interpersonal skills with patients (three items), interpersonal skills with young relatives (two items), and interpersonal skills with adult relatives (two items).

*Life Closure Skills*. Three items were used to measure this domain: the palliative care philosophy, being a human being, and living in comfort and dignity until one dies.

In the questionnaire, each of the items was rated on a 5-point Likert scale, and the questions had descriptors for all the numbers, ranging from 1 to 5, as follows: 1 = do not agree at all; 2 = disagree to some extent; 3 = agree to some extent; 4 = agree completely and 5 = not relevant [27].

### 2.3. Statistical Analysis

The quantitative data used in the study were analysed using SPSS Version 17 and Mplus Version 7 (Mplus 7). SPSS was used to check for accuracy, outliers, normality, missing data, and also internal consistency, which was measured using Cronbach's alpha. While all the items had acceptable values of skewness, a level of kurtosis above normal was found on two items measuring teamwork skills and two items measuring life closure skills. Still, according to earlier research, these four items are important aspects of clinical competence. The maximum likelihood estimation (MLE) is a rather robust method for analysis and can manage such levels of kurtosis.

Analysis of missing values revealed that the Little's missing completely at random (MCAR) test [31] was not statistically significant (chi-square = 614, 95, DF = 596, and *P* = 0.28). These results showed that the data were missing completely at random and that, consequently, it was possible to use data imputation.

Structural equation modelling (SEM) can be sensitive to the sample size [32]. Some assumptions exist concerning relationships between the theoretical concepts, and we therefore chose to run a Confirmatory factor analysis (CFA). This analytical approach builds on a theoretical framework and can provide sufficient information about residuals and the possible modifications of the relationship between the items and factors in a model.

There are several benefits to running SEM. First, the relationship between the observed factors and the latent factors is tested. Second, the relationship between the latent factors is also tested. Third, it is possible to examine the accuracy of the correlation between a hypothesised model of self-assessment and an empirical model and to get an estimation of the model fit [33]. Fourth, modification indices can be estimated in order to guide minor modifications [30].

To evaluate the fit of the instrument, a wide range of fit indices were used [32]. Brown [34] distinguished between three different fit indices: the comparative fit, an absolute fit, and a parsimony correction. Two comparative fit indices were used in this study—the comparative fit index (CFI) and the Tucker-Lewis fit index (TLI). According to Hu and Bentler [35], levels of CFI and TLI close to or above 0.95 are acceptable. A parsimony correction index can be run through a root mean square error of approximation (RMSEA). A RMSEA level below 0.08 suggests an adequate model fit while a RMSEA level below 0.06 indicates a good fit [30].

When running analysis with SEM, a chi-square test is used as a criterion [30]. A nonsignificant chi-square test will indicate whether no difference exists between the instrument and the empirical data. However, due to the chi-square test's sensitivity to sample size, the ratio of *χ* ^2^ to its degree of freedom—when *χ* ^2^/d.f. < 3—was also used in this study as a criterion of no difference between the instrument and the empirical data. Additionally, it is important that the internal structure of the instrument be of high quality, and to achieve such the levels of factor loadings have to meet the following criteria: (1) the factor loadings have to be statistically significant on a 5% level; (2) the factor loadings should be close to 0.35 or above.

### 2.4. Ethical Considerations

The study was formed on the basis of a recommendation from the management of the Lovisenberg Diaconal University College in 2007, and the college's Institutional Review Board approved the study. The study was conducted in accordance with ethical guidelines for medical research [36]. Questions that could reveal the identity of the participants were left out of the study. For example, there were fewer than 10 male students among the total study population so gender was therefore excluded.

## 3. Results

### 3.1. Characteristics of the Participants

In total 122 participants answered the questionnaire. The majority of participants (87%) have personal contact with patients. Information about the participants' workplace, years since graduation, and years of work experience as nurses are shown in Table 1.

### 3.2. Psychometric Properties: Overall Model Fit

The standardised factor loading between most of the latent variables is significant in the initial model. However, the model does not have an acceptable fit to the data because the results from the SEM analysis show that: *χ* ^2^ = 452 (d.f. = 286), *n* = 122 and *P* < 0.05, with CFI = 0.90 and RMSEA = 0.07. In order to improve the model fit, we investigated the information (in the modification output from Mplus) and found three cross loadings between the error measurements. The questions related to these error measurements were analysed in order to identify a theoretical connection between these cross loadings. One reason for accepting the cross loading was that the questions had something in common. For example, the modification indices suggested cross loading between the error measurement of dryness of mouth from the “knowledge in symptom management” variable and dryness of mouth from the “systematic use of ESAS” variable. A modified model of the instrument was converged to an acceptable solution. All parameters and relationships within the model are statistically significant (Table 2).

According to the test fit results, the chi-square of the modified model fit between the domains of the NCPC and the empirical data was found to be: *χ* ^2^ = 368 (d.f. = 283), *n* = 122, *P* < 0.05. The ratio of *χ* ^2^ to its degree of freedom is below 2, which is acceptable [35]. Examination of the other fit indices showed that the values of CFI, TLI, and RMSEA are acceptable, as follows: CFI = 0.95, TLI = 0.95, and RMSEA = 0.05 (LO 90 = 0.26 and HI 90 = 0.06).

**Figure 1:**
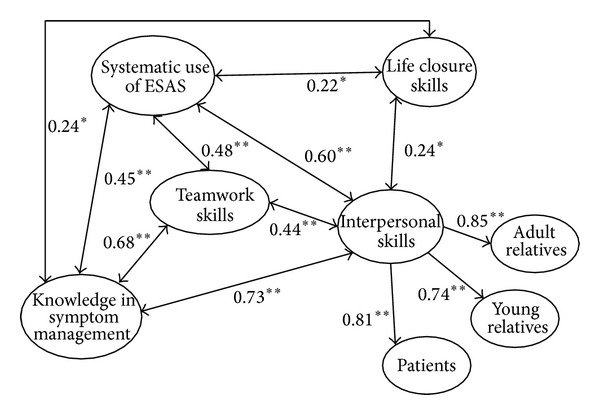
The correlation between the factors in the NCPC instrument. Only correlations above 0.20 is presented. (Note: ^*t*^ *P* < 0.1, *P < 0.05, **P < 0.01).

The results of the assessment of the quality of the internal structure of the model reveal that 26 of the 29 factor loadings, which included three factors that created second-order factors, are between 0.54 and 0.96. The three remaining factor loadings are below 0.50—two related to building interpersonal skills with patients (0.43 and 0.42) and one related to the “knowledge in symptom management” variable (0.40). The model and elements of nurses' clinical core competence in palliative care are presented in Figure 1.

## 4. Discussion

The objective of this study was to test and validate the NCPC. The validity testing showed promising results about what constitutes core competencies in clinical palliative care nursing in Norway. The construct validity seems rather good because most of the factor loadings have acceptable values, which supports the internal structure of the instrument. However, the instrument should also be tested in other countries and cultures and supplemented with new evidence about clinical competence in palliative care. The content validity of the NCPC could be evaluated using the Delphi method [37], in which an expert panel in palliative nursing could evaluate the appropriateness of the model and its usefulness in an international setting. The implications of the study's findings should be considered in the context of not only the patient's wishes but also local cultural codes in community hospitals, nursing homes, home care settings, and/or public hospitals.

In this study, the following five items were used to measure the domain “knowledge in symptom management”: dealing with pain, nausea, anxiety/restlessness, fatigue and dryness of mouth. The following six items were used to measure the domain “systematic use of ESAS”: the use of ESAS in dealing with pain, nausea, anxiety/restlessness, fatigue, dryness of mouth, and wellbeing when breaking bad news. The competencies involved with dealing with pain and dryness of mouth were given the highest scores. The competencies of hospital-based nurses in dealing with pain were significantly better than the competencies of nurses working in primary healthcare settings. The competencies had a positive effect on care routines, also seen in an earlier study [13].

In accordance with Miyashita et al. [10, 11], competence in symptom management can ensure quality end-of-life care. The importance of the systematic assessment of the care needs of palliative patients has been emphasised, and the systematic use of instruments for assessing patients' needs has been presented as one way of improving evidence-based palliative care nursing [13]. The ESAS, also called the MD Anderson symptom assessment system (ASAS), is well known. One version of the ESAS, as presented by Bruera et al. [38] and Bruera and MacDonald [39], was adapted to Norwegian conditions by the Palliative Medicine Unit at St. Olavs Hospital in Trondheim, by Loge et al. [40]. The Norwegian ESAS includes questions about pain at rest and pain during movement, fatigue, nausea, dryness of mouth, anxiety/restlessness, shortness of breath, appetite, depression, and wellbeing (e.g., “How are you feeling today?”). Questions about sleep problems, obstipation or diarrhea were not included; problems involving the oral cavity were defined as dryness of the mouth. Bergh et al. [41] examined what the ESAS responses mean and found that errors and misunderstandings do occur, which underlines the importance of examining patients' interpretations. A standardised method for using the ESAS is a prerequisite to reducing the risk of errors, which may increase the clinical utility of the instrument and improve symptom management. Norway introduced the ESAS as a standard in palliative care in 2004, and we therefore decided to include the ESAS alongside the NCPC in this study, despite parts of its content already being covered by the “knowledge in symptom management” domain. From a nursing perspective, the questions in the ESAS do not cover all the conditions that affect patients. In clinical situations, clinical decision-making depends on a nurse's ability not only to assess the severity of patients' health problems and the factors that affect patients' actual life context, but also to “know the patient.” Nevertheless, different instruments are only as good as each clinician's competence and attitude towards delivering palliative care.

In this study, the following five items were used to measure teamwork skills: collaboration in dealing with pain, nausea, anxiety/restlessness, fatigue, and dryness of mouth. Although our data are limited, they indicate that nurses with postbachelor's studies in palliative care expressed the importance of collaborating with colleagues when patients are in need of palliative care. Good collaboration might contribute to developing better care routines and better contexts for palliative care, thereby improving patient outcomes.

In palliative care, the transition towards end-of-life care might be seen as an intersection, involving the transition to hospital, returning home, and going back to hospital or hospice-like units [42]. The teamwork skills explored in our study are supported by Benner's conception of domain administering and monitoring of therapeutic interventions [43]. Patients in need of palliative care often need interorganisational care, and Hellesø et al. [44] and Hellesø and Fagermoen [45] illuminated the organisational, professional, and technological aspects that influence nurses' work. They found that nurses in different organisational contexts highlighted different information about the patients, and this might be seen as part of local cultural codes in public hospitals or in-home care. Good end-of-life care requires good collaboration and teamwork skills from all the healthcare personnel involved in delivering that care. Communication, shared common goals, and coordination between care providers are crucial for patients who need healthcare from different organisational settings.

In this study, the following three items were used to measure life closure skills: the palliative care philosophy, being a human being and living in comfort and dignity until one dies. The “life closure skills” domain is positively correlated with knowledge in symptom management but does not correlate significantly with the other domains in the instrument, that is, interpersonal skills. Life closure skills are an important part of the NCPC, yet it seems that the questions used to measure life closure skills need to be further scrutinized.

From a theoretical perspective, life closure skills are considered to be an important part of clinical competence [2, 8, 17]. The issues of life-threatening illness, dependence, and end-of-life care have a great impact on a patient's quality of life and on their families. Moreover, according to Becker [17], life closure skills are crucial to delivering high-quality care to both patients and their families, when the patient's life is close to nearing its end and even after the patient has died. The care of patients moves from hospitals to in-home care or care in nursing homes. Caring for cancer patients in the palliative phase when they are living at home seems to be more burdensome compared with caring for newly diagnosed cancer patients [46]. While Stajduhar et al. [9] have explored the context of end-of-life practice, as part of a complete understanding of the complexity of care assessment at end-of-life, more research is needed. Although life closure skills require interpersonal skills, in our study the elements of interpersonal skills focus on patients, young relatives, and adult relatives; seen here these factor loadings are good. However, when next improving the NCPC, interpersonal skills in relation to patients' families should be further tested.

In the actual study, the results of the validity testing supported the supposition that the formal, postbachelor program qualified nurses to communicate with patients and families in need of palliative care. Life closure and preparation for death are a challenge for patients and their families and nurses [6, 8, 47, 48]. Dying, after all, is not just a clinical outcome but a global human experience with real persons living in the reality of their suffering. Larkin [29] pointed out that clear evidence from research into communication skills training indicates that the failure to attend to personal attitudes does not reduce blocking behaviours if patients choose not to disclose their concerns. A limited number of studies done in the Nordic countries have included the patient's view when exploring the concept of the expert nurse in palliative care [49, 50]. There is an obvious need for more research on the effects of person-centred care and* how* to measure clinical competence in palliative care nursing. Furthermore, it seems important to continue research on the “life closure skills” domain.

## 5. Methodological Considerations

The interpretation of the results of the actual study requires the acknowledgement of several limitations. First, a more comprehensive sample size would have strengthened the study. Still, we did not find any systematic losses concerning the participants in the study. No clear, general agreement about suitable sample size exists. Researchers have provided conflicting recommendations for what should constitute the minimum number of subjects in factor analyses studies (from 100 to 400 or more) and the number of subjects per item (from five to ten), but the theoretical basis for most of these existing recommendations has been assessed as scarce [30, 51, 52]. Second, the purposive sampling that was used in the study included only those nurses who had graduated from a postbachelor, palliative care program from two different Norwegian educational institutions, which limits the generalizability of the results. Further testing, for example, in regard to specialized nurses in palliative care, is needed. Third, self-assessment is subjective and, therefore, an over- or underestimation of own achievements can occur [53–55]. It is difficult to know the degree to which self-assessed competence is related to actual behaviour, in this case nursing activities. Moreover, is it not possible to say which competencies were attributable to formal education and which were attributable to clinical experiences. A longitudinal study combining self-reporting and observations of behaviour in practice could increase insight into the validity of the instrument. We still, nonetheless, argue for the importance of having nurses describe their own perceptions of palliative competences. Instead of using a cross-sectional research design, further studies should investigate and employ other methods by gathering data from different sources and developing longitudinal research designs. Nevertheless, despite the limitations of this study, our results yielded meaningful information about nurses' core clinical competencies in palliative care.

## 6. Conclusions and Implications for Practice

The results of the study suggest that the new NCPC instrument has the potential to be used to refine clinical competence in palliative care and be used for the training and evaluation of palliative care nurses. Research is warranted to determine knowledge on core competencies in clinical palliative care in the Nordic countries. As Stajduhar et al. [9] stated, research should include the context of practice in order to develop an understanding of the complexity of care assessment at the end-of-life. We found empirical support for the NCPC, but a need exists to utilize the instrument in employee evaluations and human resource development. It will be useful to test the instrument on other samples in order to continue to evolve its utility. One current challenge is the instrument's questions on interpersonal skills. We therefore envision that in the long term the NCPC will be developed through the addition of more general questions on interpersonal skills. There are also challenges with the variable “teamwork skills,” and the analysis of consistency indicates a need for the development of more questions. The self-assessment of own clinical competence may improve awareness of nurses' strengths and weaknesses. Furthermore, an increased awareness of own competence in palliative care allows nurse managers to develop nurses' competence and lead discussions on how to develop competence in palliative care.

## Figures and Tables

**Table 1:** Information about participants' workplace, years since graduation, and years of work experience as nurses.

	*N* (%)
Personal contact with patients	106 (87%)
Workplace	
Hospital	44 (36%)
Nursing home	27 (22%)
Home service	26 (21%)
Work experience as nurses	
Less than 5 years	7 (5.7%)
Between 5 and 10 years	20 (16.4%)
More than 10 but less than 15 years	17 (13.9%)
More than 15 but less than 20 years	30 (24.6%)
More than 20 years	48 (39.3%)

**Table 2:** Internal consistencies of the latent variables and means, loadings and standard error of the items from analysis (*N* = 122, chi-square = 378, d.f. = 283, CFI = 0.95, RMSEA = 0.05, and SRMR = 0.07).

	Mean	Loading	S.E.
Life closure skills (Cronbach's alpha = 0.84)			
*v*3	3.77	0.70∗∗	0.07
*v*4	3.670	0.96∗∗	0.07
*v*5	3.65	0.57∗∗	0.07

Systematic use of ESAS (CA = 0.97)			
*v*6	3.645	0.750∗∗	0.04
*v*7	2.29	0.94∗∗	0.01
*v*8	2.70	0.96∗∗	0.01
*v*9	2.61	0.93∗∗	0.01
*v*10	2.76	0.95∗∗	0.01
*v*11	2.49	0.93∗∗	0.01

Teamwork skills (CA = 0.72)			
*v*13	3.71	0.70∗∗	0.06
*v*14	3.49	0.80∗∗	0.05
*v*15	3.60	0.74∗∗	0.05
*v*16	3.08	0.68∗∗	0.06
*v*17	3.48	0.60∗∗	0.07

Knowledge in symptom management (CA = 0.68)			
*v*22	3.41	0.54∗∗	0.08
*v*24	3.08	0.76∗∗	0.06
*v*25	3.09	0.68∗∗	0.06
*v*26	3.26	0.67∗∗	0.06
*v*27	3.37	0.40∗∗	0.09

Interpersonal skills: patients (CA = 0.57)			
*V*20	3.15	0.84∗∗	0.09
*V*21	2.98	0.43∗∗	0.10
*V*35	3.80	0.42∗∗	0.10

Interpersonal skills: young relatives (CA = 0.86)			
*V*18	2.04	0.89∗∗	0.06
*V*19	1.82	0.81∗∗	0.06

Interpersonal skills: adult relatives (CA = 0.51)			
*V*32	3.41	0.62∗∗	0.13
*V*33	3.71	0.57∗∗	0.10

Second order factor interpersonal skills			
IP patients		0.81∗∗	0.08
IP young relatives		0.74∗∗	0.12
IP Adult relatives		0.85∗∗	0.12

**P < 0.01.
